# Prevalence and factors associated with secondhand smoke exposure among Malaysian adolescents

**DOI:** 10.18332/tid/102728

**Published:** 2019-03-27

**Authors:** Sumarni Mohd Ghazali, Teh Chien Huey, Kee Chee Cheong, Lim Hui Li, Muhammad Fadhli, Mohd Yusoff, Ahmad Faudzi Yusoff, Lim Kuang Hock

**Affiliations:** 1 Epidemiology & Biostatistics Unit, Institute for Medical Research, Ministry of Health, Kuala Lumpur, Malaysia; 2 Hospital Sultan Haji Ahmad Shah, Ministry of Health, Temerloh, Malaysia; 3 Institute for Public Health, Ministry of Health, Kuala Lumpur, Malaysia

**Keywords:** secondhand smoke, adolescent, schoolchildren, smoking behavior

## Abstract

**INTRODUCTION:**

Exposure to secondhand smoke (SHS) has been proven to be detrimental to health. However, there is little information on SHS exposure among Malaysian adolescents. This study aims to assess the magnitude of and factors associated with SHS exposure among school-going adolescents in Malaysia.

**METHODS:**

We performed secondary analysis on data from 25461 respondents of the Global School Health Survey in Malaysia. Descriptive analyses and multivariable logistic regression were performed to determine factors associated with SHS exposure.

**RESULTS:**

Respondents were adolescents of mean age 14.84 (SD=1.45) years, 50.2% of which were male and 49.8% female. Approximately four in ten respondents were exposed to SHS in the past week (41.5%). SHS exposure was significantly higher among respondents who smoked than among non-smokers (85.8% vs 35.7%, p<0.001). The likelihood of exposure to SHS was higher among smoking adolescents (Adjusted OR=1.66, 95% CI: 1.07–2.56) and non-smoking adolescents (AOR=3.15, 95% CI: 1.48–4.71) who had at least one smoking parent/guardian regardless of their own smoking status. Male adolescents had higher risk of SHS exposure compared to their female counterparts (current smoker AOR=1.66, 95% CI: 1.07–2.56; non-smoker AOR=1.50, 95% CI: 1.12–2.00) and increased with age, regardless of their smoking status.

**CONCLUSIONS:**

Our findings suggest that prevalence of exposure to SHS among school-going adolescents in Malaysia is high. Parents should be advised to stop smoking or abstain from smoking in the presence of their children. Education programmes are recommended to increase awareness on avoidance of SHS as well as smoking cessation interventions for both adolescents and their parents.

## INTRODUCTION

Secondhand smoke (SHS) refers to the smoke exhaled by a smoker (mainstream smoke) and/ or the smoke released from the burning end of a cigarette (sidestream smoke)^1^. Exposure to SHS increases the risk for several diseases, such as heart disease, lung cancer, bronchitis and sudden infant death syndrome, and is especially detrimental to the respiratory development of children^2,3^. It was estimated to contribute to more than 600000 deaths among non-smokers worldwide in 2004, almost one-third of whom were children^4^. Furthermore, exposure to SHS may actually encourage adolescent smokers to continue smoking and increase the likelihood of experimental smokers becoming established or become daily smokers^5,6^. Among non-smokers, SHS exposure increases susceptibility to smoking^7,8^ and the likelihood of smoking initiation^9,10^.

Policies for the control of secondhand smoke in Malaysia began in the early 1970s, with restrictions on smoking in cinemas, followed by hospitals, clinics and health centres under the Ministries of Health and Defence, air-conditioned buses, and train coaches^11^. Then the Control of Tobacco Products Regulation 1993 (CTPR 1993) was introduced, which regulated smoking and by extension SHS. Initially, under the CTPR 1993, eight types of areas were designated as smoke-free (entertainment centres or theatres, hospitals or health clinics, public lifts, air-conditioned eateries, public transport vehicles, the Island & Peninsular building in Kuala Lumpur, petrol stations, and the Esso tower building in Kuala Lumpur). The list of areas was subsequently expanded to comply with Article 8 of the Framework Convention on Tobacco Control, and continues to expand. As of 2018, the law covers up to 29 types of public areas and nine localities including schools and other educational institutions. However, a study conducted in 61 hospitality venues, two-thirds of which were supposedly smoke-free, reported high levels of SHS^12^ even in the smoking prohibited areas, which suggested poor enforcement of and compliance with the law. With better enforcement, the existing policies may successfully reduce SHS in public spaces, however, though adolescents may be protected from SHS exposure in schools and other public places, they may still be exposed to SHS in their homes. The Global Youth Tobacco Survey, which was conducted in 168 countries, reported that about 47% of non-smokers are exposed to SHS at home and around 48% are exposed to SHS outdoors^13^.

Almost a quarter of Malaysian adults are smokers^14,15^, thus exposure of Malaysian adolescents to SHS is suspected to be substantial. Thus far, there have only been two studies conducted among adolescents in Malaysia. One of the studies that was conducted in Peninsular Malaysia involving 2599 students found 56.4% prevalence of SHS exposure^15^. The other study was localized in Kuala Lumpur and Negeri Sembilan and found 66.9% self-reported SHS exposure among 695 primary school students aged 12 years or younger^16^. We conducted a study using data from a nationwide survey (Malaysian Global School-based Student Health Survey 2012) to describe the prevalence and factors associated with SHS exposure among adolescents in Malaysia, in view of the importance of these data in policy formulation and towards improving existing policies on SHS with regards to adolescents.

## METHODS

The data for this study were derived from the 2012 Malaysia Global School-based Student Health Survey (GSHS-M). The GSHS-M methodology has been described in detail elsewhere (Yusoff et al.^17^). In brief, the GSHS-M sample was selected using two-stage stratified cluster sampling to obtain a representative sample of secondary school-going adolescents (age 12–17 years), using a sampling frame provided by the Ministry of Education, Malaysia. The first stage was selection of secondary schools and the second stage was selection of classes from the selected schools based on the size of enrolment. All students from the selected classes were invited to participate in the study but only those with written informed consent from parents/ guardians were enrolled.

The study instrument was adapted from the Global School-based Student Survey questionnaire developed by the World Health Organization^18^, which was translated into the Malay language and validated by a panel of experts. Before questionnaire administration, trained research team members explained to the participants the objectives of the study, voluntary participation and explained each item in the questionnaire. The questionnaire was then self-administered by the participants.

Teachers and school staff were not allowed in the room while the students were filling in the questionnaires to avoid the ‘Hawthorne effect’. Completed questionnaires were collected and sealed in an opaque envelope in front of the students. The study protocol was approved by the Ministry of Education, whereas the state and district education departments and the administrators of the respective schools approved the recruitment of the participants. The study was vetted and approved by both the ethics committees of the Ministry of Health and the Ministry of Education of Malaysia.

### Measurements

The dependent variable ‘exposure to SHS’ was self-reported and measured by the item ‘During the past seven days, on how many days did people smoke in your presence?’. Students could respond with ‘0 days’, ‘1–2 days’, ‘3–4 days, ‘5–6 days’ or ‘All 7 days’. We defined ‘Exposed to SHS’ as self-reported exposure on at least one day in the past seven days, and ‘Not exposed to SHS’ as 0 days of exposure in the past seven days. The independent variables examined were gender, age (using the proxy variable form), smoking status and parental smoking status. Smoking status comprised two categories: current smoker (smoked at least once in the past 30 days) or non-smoker (did not smoke at all in the past 30 days).

### Statistical analysis

The data were cleaned and weights were applied in the statistical analysis to account for the complex survey design and response rate to ensure that estimated proportions were representative of the secondary school-going adolescents’ population of Malaysia. The sociodemographic characteristics of the sample and the prevalence of SHS exposure were described in frequencies, percentages and estimated population. Chi-squared analysis was used to test for crude association between the independent variables and SHS exposure. Multiple logistic regression was used to identify variables independently associated with SHS exposure and to obtain adjusted odds ratios and their respective 95% confidence intervals. There were significant two-way interactions between smoking status and gender, and between smoking status and age. Therefore, the multiple logistic regression model was stratified by smoking status and two separate models were generated, one for smokers and another for non-smokers. All statistical analyses were carried out at 95% significance level using the complex samples module in SPSS statistics software version 22.

## RESULTS

The total number of students who were eligible was 28998. The number of participants in the GSHS-M study was 25461 (response rate of 87.8%). The remaining 12.2% did not respond due to absence from school, parents did not consent, or absent from class due to other extra-curricular school activities. Respondents were between 13–17 years of age (Mean=14.84, SD=1.45). Male and female respondents were approximately equally distributed. Of the 25461 respondents, 12% (2836/25461) were current smokers. A majority of respondents reported that their parents/guardians did not smoke (59.8%) (Table 1).

**Table 1:** Characteristics of the GSHS-M respondents (n=25461 )

*Variable*	*Estimate*	*n*	*% ( 95% CI)*
**Gender**			
Male	1126613	12732	50.2 (49.4–50.9)
Female	1118911	12729	49.8 (49.1–50.6)
**Age (years)**			
13	490521	5433	21.9 (21.2–22.5)
14	458559	5329	20.4 (19.8–21.7)
15	449942	5599	20.1 (19.5–20.6)
16	431501	4515	19.2 (18.6–19.9)
17	416457	4571	18.4 (17.8–19.0)
**Smoking status**			
Yes	269079	2836	12.0 (11.5–12.5)
No	1981135	22671	88.0 (87.5–88.5)
**At least one parent/guardian smoked**			
Yes	901410	10262	40.2 (39.5–40.9)
No	1340356	15150	59.8 (59.1–60.5)

CI: confidence interval

**Figure 1:**
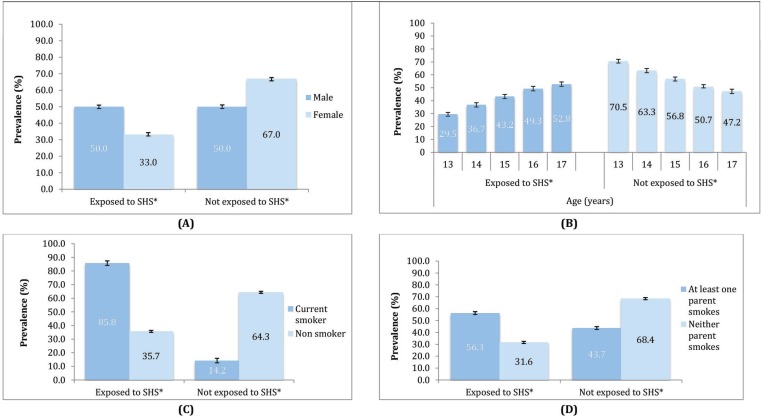
Prevalence of exposure to secondhand smoke (SHS) among GSHS-M respondents by (A) Gender, (B) Age, (C) Self-smoking status, (D) Parents’/guardians’ smoking status

The overall prevalence of SHS exposure was 41.5%. SHS exposure was significantly higher among male (50%, 95% CI: 48.9–51.0) compared to female students (33.0%, 95% CI: 32.3–34.3). The prevalence of exposure to SHS among current smokers (85.8%) was almost 2.5 times the rate of exposure among non-smokers (35.7%). Exposure to SHS was higher among respondents whose parents or guardians smoked and the prevalence of exposure increased in a linear fashion from age 13 to 17 years (Figure 1).

Multivariable analysis showed that male, older aged and having at least one smoking parent or guardian were independent risk factors for SHS exposure, regardless of the adolescent’s smoking status (Table 2).

**Table 2:** Multiple logistic regression for the association between sociodemographic factors and SHS exposure in the GSHS-M study

*Variable*	*Non-smoker (n=22502)*		*Current smoker (n=2658)*	
	*AOR*	*95% CI*	*AOR*	*95% CI*
**Gender**				
Male	1.50	1.12–2.00	1.66	1.07–2.56
Female	1		1	
**Age (years)**				
13	1		1	
14	1.37	1.23–1.54	2.21	1.43–3.40
15	1.76	1.58–1.96	2.78	1.85–4.20
16	2.03	1.82–2.27	4.02	2.93–7.28
17	2.59	2.31–2.89	4.01	2.48–6.50
**Parental smoking status**				
At least one parent smoked	3.15	1.48–4.71	1.66	1.07–2.56
Neither parent smoked	1		1	

AOR: adjusted odds ratio. CI: confidence interval

## DISCUSSION

The SHS exposure rate of 41.5% in this study is very similar to the 2004 global estimated prevalence of 40%^4^ among children and 43% among adolescents in Nigeria^19^. However, our rate is more than twice as high as the prevalence of SHS exposure among adolescents in Africa (12.5%)^20^ and Germany (18.8%)^21^. Another Malaysian study reported a higher prevalence of 52.9% SHS exposure at home based on salivary cotinine measurement^16^. A study among Greek adolescents aged 12–18 years^22^ reported 59.1% prevalence. In addition, the prevalence in this study is 15% lower than that reported in 68 low- and middle-income countries (55.9%)^23^, Cambodia (67.1%)^24^ and Mongolia (73.9%, 71.6–76.1%, males; and 71.7%, 69.7–73.7% females)^25^. The varying prevalences of adolescents with SHS exposure may be attributed to variations in sociodemographic characteristics, prevalence of smoking among adults and adolescents, community norms and legislation regarding tobacco control (e.g. duration and level of enforcement) between countries.

In this study, exposure to SHS was higher among smokers, consistent with the findings of other studies locally and abroad^15,26^. The findings are consistent with a previous study in upper secondary school going adolescents in Peninsular Malaysia in which the level of exposure to SHS among smokers was almost twice as high as those who did not smoke^15^.

Similar findings were reported from studies among high school students in the US^27^ and South Africa^28^. This may be because smokers tend to befriend other smokers as they share a common behaviour that is smoking^29^. A previous local study found that more than 90% of adolescents tend to smoke together in groups^30^. These factors increase smokers’ exposure to SHS. On the other hand, non-smoking adolescents tend to avoid smokers, thus reducing their exposure to SHS. Additionally, adolescents who do not smoke may have better knowledge of the hazardous effects of SHS and are therefore more keen to avoid SHS exposure than those who smoke^31^, thus explaining their lower likelihood of SHS exposure.

Exposure to SHS was high among male respondents regardless of smoking status. These findings are contrary to a US study that found higher susceptibility to SHS exposure among adolescent girls^27^, while another Malaysian study found no relationship between gender and exposure to SHS^15^. This may be due to differences in the prevalence of smoking among adolescents in the USA where there is no difference in smoking prevalence between gender, while in Malaysia the prevalence of female smoking is very low. Whereas the study by Lim et al.^30^ involved students aged between 16–17 years, in the current study the age range was wider. Thus, differing sociodemographics may be one of the reasons for the difference. The higher likelihood of exposure to SHS among male smokers may be due to adolescent male smokers tending to smoke as a group^30^. We postulate that the lower likelihood of SHS exposure among females is due to the prevailing social norms against female smoking, especially teenage girls, which encourage girls to smoke in private. The same pattern is also observed among non-smokers, where SHS exposure was found to be higher among non-smoking boys compared to non-smoking girls.

We found a positive relationship between age and exposure to SHS irrespective of smoking status. This is in line with studies in Cambodia^24^, Mongolia^25^, Taiwan^31^ and United States^27^. In addition, this finding is consistent with Veeranki et al.^13^ who found an increase in prevalence of exposure to SHS from 21.2% among those aged 13 years, to 24.0% and 24.6% among those aged 14 years and 15 years, respectively. This may be due to older adolescents being allowed more freedom by their parents given their higher level of maturity, therefore they are more mobile and able to frequent places such as non-air-conditioned bistros and coffee houses where smoking is prevalent^11^, which may increase their likelihood for exposure to SHS. Parents are more concerned about younger teenagers’ health and safety, and thus exert more control over their movements^30^ limiting their exposure to health hazards such as SHS. Furthermore, the prevalence of smoking has been shown to be lower among younger participants^32^ who have less social networking with peers, that may also explain why they are less exposed to SHS^33^. However, a study in Nigeria^19^ reported an inverse relationship between age group and likelihood of exposure to SHS. Peltzer et al.^28^ reported no significant association between age and exposure to SHS at home or outside home in South Africa. However, it should be noted that there is a marked difference in the age of participants between studies. The previous studies’ target populations were adolescents aged 11 to 18 years while the present study only focused on those aged 13 to 17 years. Therefore, the magnitude of difference in SHS exposure in the present study was lower than in previous studies that had a relatively wider age range.

We found that adolescents who had at least one smoking parent were more likely to be exposed to SHS. Other studies in South Africa^28^, Korea^33^ and Mexico^34^ as well as the Global Youth Tobacco Survey (GYTS)^13^ showed similar findings. The association may be explained as follows: If a parent or both parents smoke, especially at home, it is unlikely that the spouse or other members of the household will stop them although they disapprove of it in order to avoid conflict, especially if the smoker is the head of the family, elderly or male^35,36^. This is because males and the elderly have higher social status in the family and their actions are not likely to be challenged^36^. The shrinking of public spaces where smoking is allowed, due to a widening of smoking prohibited areas in Malaysia under the amended Control of Tobacco Product Regulation 2004, has made it more likely for them to smoke at home^37^. Parents or guardians who smoke may also be more lenient towards SHS and are unlikely to prohibit guests or household members from smoking inside the house, compared to their non-smoking counterparts. The Global Adult Tobacco Survey (GATs) among Malaysian adults reported only 22% of adult smokers impose a total smoking ban at home compared to 46.9% of non-smokers^11^. This indicates that SHS exposure among adolescents is positively associated with the prevalence of smoking among adults suggesting that more aggressive anti-smoking measures targeting the smoking adult population should be initiated by the government to protect adolescents against the harmful effects of SHS.

### Limitations

This study has several limitations. Exposure to SHS was self-reported and not verified through salivary or serum cotinine measurement, therefore there may have been imperfect recall of SHS exposure. Also, independent variables that have been shown to be significant in other studies, such as smoking status of household members other than the parents or guardians, smoking status of peers, attitude towards smoking and knowledge of the associated hazards of SHS, socioeconomic status, restriction of smoking at home (total, partial or no restriction), were not measured in the GSHS-M, and thus not accounted for in the current study. Location of exposure to SHS (whether exposure occurred at home or other places) was also not determined. Therefore, an opportunity was missed to determine suitable measures that can be recommended to address SHS exposure in different settings. Further studies might consider investigating location of exposure. However, this study was based on a large, representative sample with high response rate, which enables the generalization of the results to the school-going adolescent population in Malaysia.

## CONCLUSIONS

An estimated 932451 Malaysian adolescents (41.5%), were exposed to SHS and those who were male, older and have smoking parent(s) or guardian(s) were more likely to be exposed to SHS. Future research should explore the possibility of enacting laws that prohibit parents/ guardians who smoke from exposing their children to secondhand smoke. Future studies should incorporate an objective measurement for SHS, quantify the intensity, frequency and the location of SHS exposure, knowledge, attitude and practice regarding SHS. Public health personnel or educators are recommended to conduct education programmes to raise awareness and avoidance of SHS for adolescents and their parents, as well as smoking cessation interventions for both.

## References

[1] International Agency for Research on Cancer (2004). Tobacco smoke and involuntary smoking. IARC Monographs on the Evaluation of Carcinogenic Risks to Humans.

[2] Janson C, Chinn S, Jarvis D, Zock JP, Toren K, Burney P (2001). Effect of passive smoking on respiratory symptoms, bronchial responsiveness, lung function, and total serum IgE in the European Community Respiratory Health Survey: a cross-sectional study. Lancet.

[3] US Department of Health and Human Services (2006). The Health Consequences of Involuntary Exposure to Tobacco Smoke: A Report of the Surgeon General.

[4] Oberg M, Jaakkola MS, Woodward A, Peruga A, PrussUstun A (2011). Worldwide burden of disease from exposure to second-hand smoke: a retrospective analysis of data from 192 countries. Lancet.

[5] Darling H, Reeder AI, McGee R, Williams S (2006). Brief report: Disposable income, and spending on fast food, alcohol, cigarettes, and gambling by New Zealand secondary school students. J Adolesc.

[6] Seo DC, Torabi MR, Weaver AE (2008). Factors influencing openness to future smoking among nonsmoking adolescents. J School Health.

[7] Lessov-Schlaggar CN, Wahlgren DR, Liles S (2011). Sensitivity to secondhand smoke exposure predicts future smoking susceptibility. Pediatrics.

[8] Okoli CT, Kodet J (2015). A systematic review of secondhand tobacco smoke exposure and smoking behaviors: Smoking status, susceptibility, initiation, dependence, and cessation. Addic Behav.

[9] Abidin NZ, Zulkifli A, Abidin EZ (2014). Secondhand smoke exposure in toddlerhood and cognitive ability among Malaysian adolescents. Iran J Public Health.

[10] Wang MP, Ho SY, Lam TH (2011). Parental smoking, exposure to secondhand smoke at home, and smoking initiation among young children. Nicotine Tob Res.

[11] Institute of Public Health (IPH) (2012). Report on Global Adult Tobacco Survey (GATS): Malaysia, 2011.

[12] Abidin EZ, Hashim Z, Semple S (2013). Second-hand smoke in public spaces: How effective has partial smoke-free legislation been in Malaysia?. Asian Pac J Cancer Prev.

[13] Veeranki SP, Mamudu HM, Zheng S (2015). Secondhand smoke exposure among never-smoking youth in 168 countries. J Adolesc Health.

[14] Institute for Public Health (IPH) (2015). National Health and Morbidity Survey 2015 (NMHS 2015). Vol II: Noncommunicable diseases risk factors & other health problems.

[15] Lim HL, Teh CH, Kee CC, Sumarni MG, Sayan P, Lim KH (2018). Exposure to second-hand smoke among secondary school-going adolescents: Findings from the Malaysian Adolescent Health Risk Behaviour (MyAHRB) study. Proceedings of Singapore Healthcare.

[16] Abidin EZ, Semple S, Omar A, Rahman HA, Turner SW, Ayres JG (2011). A survey of schoolchildren’s exposure to secondhand smoke in Malaysia. BMC Pub Health.

[17] Yusoff F, Saari R, Naidu BM, Ahmad NA, Omar A, Aris T (2014). Methodology of the National School-based Health Survey in Malaysia, 2012. Asia Pac J Public Health.

[18] World Health Organization Global School-based Student Health Survey (GSHS 2009).

[19] Omaduvie U, Adisa A (2015). Exposure to secondhand smoke in the home and public areas among adolescents in Abuja, Nigeria: Tobacco control implications. Tob Prev Cessation.

[20] Owusu D, Mamudu HM, John RM, Ibrahim A, Ouma AE, Veeranki SP (2016). Never-smoking adolescents’ exposure to secondhand smoke in Africa. Am J Prev Med.

[21] Kuntz B, Lampert T (2016). Smoking and passive smoke exposure among adolescents in Germany. Deutsches Ärzteblatt International.

[22] Lappas AS, Tzortzi AS, Konstantinidi EM, Dimou N, Behrakis PK (2015). Factors associated with exposure to passive smoking among 12-18 year-old students in Athens and Thessaloniki, Greece. Tob Prev Cessation.

[23] Xi B, Liang Y, Liu Y (2016). Tobacco use and secondhand smoke exposure in young adolescents aged 12-15 years: data from 68 low-income and middle-income countries. The Lancet Global Health.

[24] Rudatsikira EM, Knutsen SF, Job JS (2008). Exposure to environmental tobacco smoke in the nonsmoking population of Cambodia. Am J Prev Med.

[25] Rudatsikira E, Siziya S, Dondo J, Muula AS (2007). Prevalence and correlates of environmental tobacco smoke exposure among adolescents in Mongolia. Indian J Pediatr.

[26] Jallow IK, Britton J, Langley T (2018). Prevalence and factors associated with exposure to secondhand smoke (SHS) among young people: a cross-sectional study from the Gambia. BMJ Open.

[27] Agaku IT, Vardavas CI (2013). Disparities and trends in indoor exposure to secondhand smoke among U.S. adolescents: 2000-2009. PLoS ONE.

[28] Peltzer K (2011). Determinants of exposure to second-hand tobacco smoke (SHS) among current non-smoking in-school adolescents (aged 11-18 years) in South Africa: results from the 2008 GYTS study. Int J Environ Res Public Health.

[29] Michell L (1997). Loud, sad or bad: young people’s perceptions of peer groups and smoking. Health Educ Res.

[30] Lim KH, Amal NM, Hanjeet K (2006). Prevalence and factors related to smoking among secondary school students in Kota Tinggi District, Johor, Malaysia. Trop Biomed.

[31] Li MF, Wang RH (2006). Factors related to avoidance of environmental tobacco smoke among adolescents in southern Taiwan. J Nurs Res.

[32] Institute of Public Health (IPH (2016). Tobacco and E-Cigarette Survey among Malaysian Adolescents (TECMA.

[33] Hwang JH, Park SW (2016). Sex and age differences in exposure to secondhand smoke at home among Korean adolescents: A nationally representative survey. Int J Environ Res Public Health.

[34] Bird Y, Moraros J, Olsen LK, Coronado GD, Thompson B (2006). Adolescents’ smoking behaviors, beliefs on the risks of smoking, and exposure to ETS in Juarez, Mexico. Am J Health Behav.

[35] Mao A (2013). Space and power: young mothers’ management of smoking in extended families in China. Health Place.

[36] Ooi J, Teh K, Tam C, Sadasivan S, Kadirvelu A (2014). Passive smoking: Perceptions and practices among urban working adults. Int J Collab Res Internal Med Public Health.

[37] Ho SY, Wang MP, Lo WS (2010). Comprehensive smoke-free legislation and displacement of smoking into the homes of young children in Hong Kong. Tob Control.

